# Novel Nomograms-Based Prediction Models for Patients with Primary Undifferentiated Pleomorphic Sarcomas Resections

**DOI:** 10.3390/cancers13081917

**Published:** 2021-04-15

**Authors:** Qiaowei Lin, Qiuyi Huang, Qifeng Wang, Wangjun Yan, Yangbai Sun

**Affiliations:** 1 Shanghai Cancer Center, Department of Musculoskeletal Surgery, Fudan University, Shanghai 200030, China; 17211210041@fudan.edu.cn (Q.L.); huangqiuyi929@sina.com (Q.H.); 2 Shanghai Cancer Center, Department of Pathology, Fudan University, Shanghai 200030, China; Wangqifeng19821982@126.com

**Keywords:** undifferentiated pleomorphic sarcomas (UPS), prognosis, nomogram

## Abstract

**Simple Summary:**

Undifferentiated pleomorphic sarcomas (UPS) are one of the most common soft tissue sarcomas which have relatively high potentials of recurrence and metastasis. Surgery remains the mainstream treatment for UPS patients. However, in modern medicine, doctors nowadays lack proper models to tell patients the exact prognosis of individuals after they have undergone primary surgery. In this work, we for the first time develop two nomograms that are able to predict 3- and 5-year overall survival (OS) and time to recurrence (TTR) for UPS patients. These nomograms show relatively good accuracy and practicability which may contribute a lot to the modern medical decision-making process.

**Abstract:**

Background: Undifferentiated pleomorphic sarcomas (UPS) were one of the most common soft tissue sarcomas. As UPS had relatively high potentials of recurrence and metastasis, we designed two nomograms to better predict the overall survival (OS) and time to recurrence (TTR) for patients who underwent primary surgery. Methods: The data of UPS patients who underwent primary surgery were extracted from Shanghai Cancer Center, Fudan University. Multivariate analyses were performed using Cox proportional hazards regression to identify independent prognostic factors. Kaplan–Meier analysis was used to compare differences for patients who underwent primary surgery in OS and TTR. Nomograms were designed with the help of R software and validated using calibration curves and receiver operating characteristic curves (ROC). Results: Kaplan–Meier curves showed that patients with older ages (*p* = 0.0024), deeper locations (*p* = 0.0422), necrosis (*p* < 0.0001), G3 French Federation Nationale des Centres de Lutte Contre le Cancer (FNCLCC) classification (*p* < 0.0001), higher Ki-67 (*p* < 0.0001), higher mitotic index (*p* < 0.0001), R1/R2 resections (*p* = 0.0002) and higher invasive depth (*p* = 0.0099) had shorter OS than the other patients while patients with older ages (*p* = 0.0108), necrosis (*p* = 0.0001), G3 FNCLCC classification (*p* < 0.0001), higher Ki-67 (*p* = 0.0006), higher mitotic index (*p* < 0.0001) and R1/R2 resections (*p* < 0.0001) had shorter TTR compared with those without. Multivariate analyses demonstrated that mitotic rates and surgical margin were independent factors for TTR while age and invasive depth were independent factors for OS. Three parameters were adopted to build the nomograms for 3- and 5-year OS and TTR. The Area Under Curve (AUC) of this nomogram at 3- and 5-year TTR reached 0.802, 0.814, respectively, while OS reached 0.718, 0.802, respectively. Calibration curves for the prediction of 3- and 5-year OS and TTR showed excellent agreement between the predicted and the actual survival outcomes. Conclusions: Some important parameters could be used to predict the outcome of individual UPS patients such as mitotic age, rates, surgical margin, and invasive depth. We developed two accurate and practicable nomograms that could predict 3- and 5-year OS and TTR for UPS patients, which could be involved in the modern medical decision-making process.

## 1. Introduction

Soft tissue sarcomas (STS), as a heterogeneous group of rare malignancies with over 50 biologically and clinically distinct types described by the World Health Organization (WHO) [1], accounted for approximately 1% of all adult malignancies [2,3]. Undifferentiated pleomorphic sarcomas (UPS), which used to be known as malignant fibrous histiocytomas (MFH), were one of the most common types of adult STS and they accounted for nearly 10% of adult STS [4,5]. Despite long history usage of the term MFH, the WHO classifications of STS finally considered the term a misnomer because they encompassed the morphologic manifestations of a variety of poorly-differentiated tumors [6]. The diagnosis of UPS was often considered when sarcomas had no distinct differentiations based on careful histological examinations and ancillary techniques, including immunohistochemistry (IHC) and molecular biology [7,8,9]. However, nobody demonstrated the exact differentiation origins of UPS cells. High levels of structural variants but a relative paucity of single nucleotide variants was confirmed in UPS by modern sequencing [10,11].

For localized STS, surgical resections, usually accompanied by radiation, remained the mainstay treatment options [12,13,14]. However, the role of adjuvant chemotherapy in STSs was still controversial [15], especially in UPS. Movva S et al. found that overall survival (OS) was improved in the chemotherapy group, and this result remained significant even after adjustment with propensity score weighting [16]. However, the largest trial of adjuvant chemotherapy by the European Organization for Research and Treatment of Cancer (EORTC) Soft Tissue and Bone Sarcoma Group with doxorubicin and ifosfamide failed to reach any significant differences in disease-free survival (DFS) or OS [17]. STS were generally considered to be poorly immunogenic tumors and had low mutational burdens [10], but pembrolizumab demonstrated promising activity in patients with advanced sarcomas among participants of SARC028, especially in UPS and dedifferentiated liposarcoma (DDLPS), with 40% and 20% of patients achieving objective response, respectively [18]. Despite the administration of radical surgical resection, up to 40% of patients would develop metastatic disease, mainly in lung [19]. A total of 30–50% of UPS patients died within 5 years after they had been diagnosed [20]. Adverse prognostic factors for UPS included large size, deep-seated location, positive surgical margins, lower-extremity location, local recurrence and metastases at presentation [21]. However, as far as we were concerned, no nomogram had been built for UPS patients treated with surgery on the basis of population-based data. Therefore, we aimed to build the first two nomograms for predicting overall survival (OS) and time to recurrence (TTR) for UPS patients who underwent primary surgery based on population-based data in our center.

## 2. Materials and Methods

### 2.1. Patients

UPS patients who underwent radical resections at Shanghai Cancer Center, Fudan University between 2006 to 2017 were retrospectively reviewed and enrolled in this study (*n* = 116). The inclusion criteria for the eligible patients were as follows: (1) primary surgery; (2) histologically diagnosed as UPS; (3) no secondary tumor; (4) complete clinicopathologic information and follow-up information. Informed signed written consent was obtained from each patient. Ethical approval was obtained from the Research Ethics Committee of Shanghai Cancer Center (FUSCC-IACUC-S20210388).

### 2.2. Pathological Examinations

The diagnosis was confirmed by a pathologist specialized in STS in our center. The following pathological features were recorded: primary tumor location, tumor size, incisal margin, necrosis, invasive depth, mitosis rate, Ki67, and French Federation Nationale des Centres de Lutte Contre le Cancer (FNCLCC) classification.

### 2.3. Follow-up Data

Follow-up data were collected by telephone and electronic medical records of our center. All 116 patients were followed up to either December 2019 or the date of death or recurrence. One of the primary endpoints in this study was OS, which was defined as the time from the date of surgery to the date of death or last follow-up time. The other primary endpoint was TTR, which was defined as the time from the date of surgery to the date of recurrence or last follow-up time.

### 2.4. Statistical Analysis

Statistical analyses were performed using the IBM SPSS Statistics 23 (SPSS Inc., Armonk, NY, USA) and GraphPad Prism 7.0 (GraphPad Software, La Jolla, CA, USA) software. Multivariate analyses were performed using Cox proportional hazards regression to identify independent prognostic factors. Kaplan–Meier analysis was used to compare differences for patients who underwent curative surgery in OS and TTR. A *p*-value < 0.05 was considered statistically significant.

### 2.5. Construction of the Nomograms

The R package ‘survival’ was used to figure out independent prognostic factors by using the univariate and multivariate Cox regression analyses. Independent prognostic factors were incorporated to construct the nomograms. In our outcomes, although surgical margins and age did not reach statistical significance in OS and TTR, respectively, their *p*-values were close to 0.05 and they had been confirmed to be independent prognostic factors in Domagoj’s study [21]. Therefore, they were adopted to optimize the nomograms. By combining these factors, we built the nomograms for 3- and 5-year OS and TTR in UPS patients with the help of R package ‘rms’. To assess the predictive accuracy of the nomograms, we performed receiver operating characteristic curves (ROC) analysis by R package ’timeROC’. Calibration curves were generated to visualize the discriminations between actual 3- and 5-year and predicted OS or TTR using R package ‘rms’. The match of calibration curves and the 45-degree line represented a perfect accuracy between the nomogram-predicted survival (*X*-axis) and actual survival (*Y*-axis).

### 2.6. Elaboration of the Nomograms and Calibration Curves

**Figure 1:**
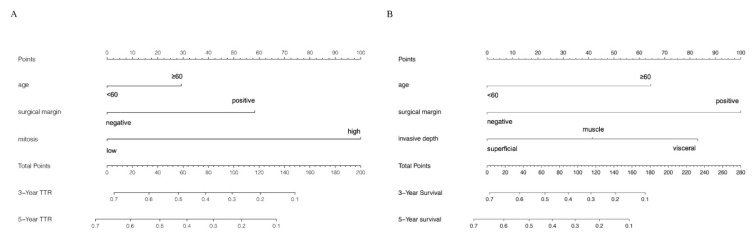
Nomograms for predicting 3- and 5-year (**A**) TTR and (**B**) OS for UPS patients who underwent primary surgery resections. Total points were calculated through addition of the scores of each clinicopathological feature mentioned above. The 3-year and 5-year possibility of recurrence (**A**) or survival (**B**) were obtained by drawing a vertical line from total points to 3-year and 5-year TTR or OS.

According to the attribution of each variate to the outcomes in multivariate regression model (the value of the regression coefficient), each value of each variate was scored. Then, each score was added to obtain the total score. Finally, the predicted probability of the individual outcome was calculated through the functional transformation relationship between the total score and the occurrence probability of the outcome event. Take our OS nomogram for an example, if a patient had a primary surgical resection of UPS with the following features, (1) age = 70, (2) negative surgical margin, (3) muscle infiltration, then each feature had 65, 0 and 40 points, respectively. We obtained the total points of 105. Then, we looked at point 105 on the total points, and we drew a vertical line to the 3-year survival and 5-year survival lines. The corresponding points were the possibility of the patient’s 3-year and 5-year survival (Figure 1).

As for the calibration curves, the 45-degree line represents a perfect match between the nomogram-predicted survival (*X*-axis) and actual survival (*Y*-axis). The perpendicular line represents 95% confidence intervals of actual survival. The little bars above 1.0 actual values represented numbers of patients in a certain range.

### 2.7. Construction of Risk Stratification System

The risk score was calculated as follows: risk score = exp_factor1_ × β_factor1_ + exp_factor2_ × β_factor2_ + exp_factorn_ × β_factorn_, where exp was the value of independent prognostic factors and β was the regression coefficient derived from the multivariate cox regression analysis. A median risk score was used as cut-off criteria to classify patients into high-risk or low-risk patients and Kaplan–Meier curves were performed to compare their differences OS and TTR. All analyses were conducted using R software version 3.4.3. Differences were considered significant at a two-sided *p*-value < 0.05.

## 3. Results

### 3.1. Patient Characteristics

Comprehensive clinical characteristics of 116 UPS patients who underwent primary surgeries in Shanghai Cancer Center, Fudan University between 2006 and 2017 are shown in Table 1. Of those patients, the median age was 57.9 years (range 17–91 years) with a female-to-male ratio of approximately 1:1. UPS most commonly occurred in extremities and trunks (*n* = 89), then came abdomen and other sites (*n* = 27). Most patients had tumors larger than 5 cm (*n* = 85), probably because older patients in China paid less attention to their physical changes and cared more about their expenses as they had just ensured their basic living needs. The median tumor size was 8.27 cm (range 1.5–30 cm). Necrosis could be seen in 53 patients. With regard to the invasive depth, most tumors infiltrated the muscles (*n* = 72), followed by viscera (*n* = 30) and superficial tissues (*n* = 14). According to the FNCLCC grading systems, 84 patients were classified as grade 3, whereas 32 patients were classified as grades 1 and 2. R0 resections were performed in 95 cases while the other 21 patients underwent R1 or R2 resections in our center. At the end of follow-up, 75 patients experienced the outcomes of local recurrence or distant metastasis and 51 patients died from UPS or other reasons.

### 3.2. Kaplan–Meier Curves of TTR and OS

**Figure 2:**
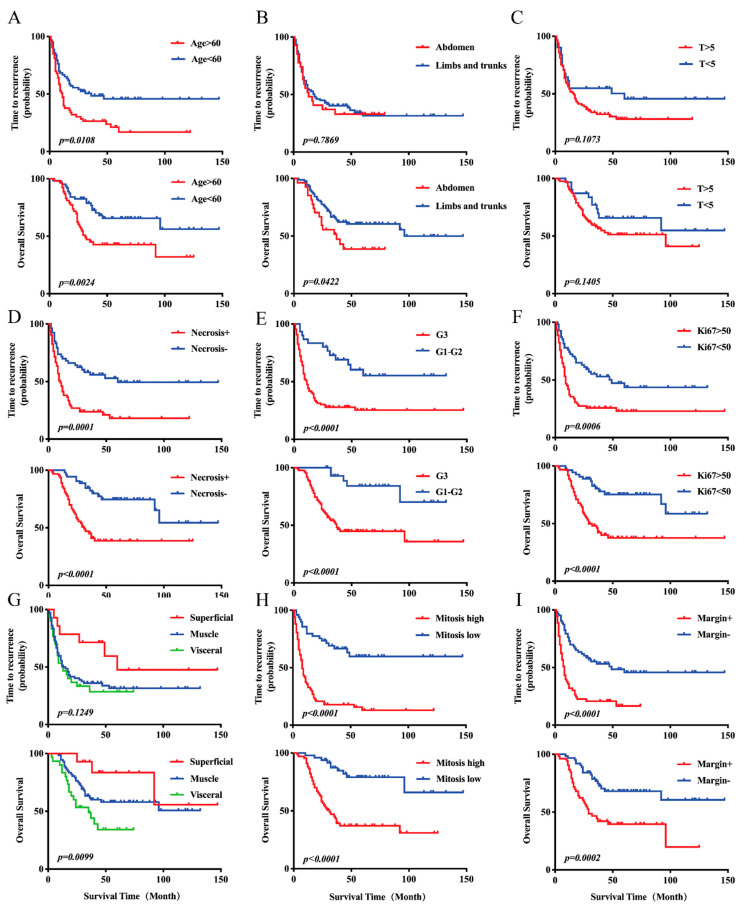
Prognostic significance of clinicopathological factors in UPS patients (*n* = 116). (**A**) Kaplan–Meier analysis of time to recurrence (TTR) (upper) and overall survival (OS) (bottom) according to age. (**B**) Kaplan–Meier analysis of TTR (upper) and OS (bottom) according to tumor site. (**C**) Kaplan–Meier analysis of TTR (upper) and OS (bottom) according to tumor size (cm). (**D**) Kaplan–Meier analysis of TTR (upper) and OS (bottom) according to necrosis. (**E**) Kaplan–Meier analysis of TTR (upper) and OS (bottom) according to French Federation Nationale des Centres de Lutte Contre le Cancer (FNCLCC) grade. (**F**) Kaplan–Meier analysis of TTR (upper) and OS (bottom) according to Ki-67. (**G**) Kaplan–Meier analysis of TTR (upper) and OS (bottom) according to invasive depth. (**H**) Kaplan–Meier analysis of TTR (upper) and OS (bottom) according to mitosis. (**I**) Kaplan–Meier analysis of TTR (upper) and OS (bottom) according to surgical margin.

Kaplan–Meier curves were performed to evaluate the prognostic values of clinicopathologic factors in UPS patients. We observed that patients with older ages (median TTR, 23 months vs. 36 months, *p* = 0.0108), necrosis (median TTR, 20 months vs. 42 months, *p* = 0.0001), G3 FNCLCC classification (median TTR, 22 months vs. 53 months, *p* < 0.0001), higher Ki-67 (median TTR, 21 months vs. 40 months, *p* = 0.0006), higher mitotic index (median TTR, 18 months vs. 46 months, *p* < 0.0001) and R1/R2 resections (median TTR, 12 months vs. 34 months, *p* < 0.0001) had shorter TTR compared with those without (Figure 2A,D–F,H,I, upper curves). As for OS, patients with older ages (median OS, 40 months vs. 50 months, *p* = 0.0024), deeper locations (median OS, 37 months vs. 48 months, *p* = 0.0422), necrosis (median OS, 36 months vs. 57 months, *p* < 0.0001), G3 FNCLCC classification (median OS, 38 months vs. 66 months, *p* < 0.0001), higher Ki-67 (median OS, 36 months vs. 56 months, *p* < 0.0001), higher mitotic index (median OS, 37 months vs. 57 months, *p* < 0.0001), R1/R2 resections (median OS, 30 months vs. 49 months, *p* = 0.0002) and higher invasive depth (median OS, 31 months vs. 48 months vs. 62 months, *p* = 0.0099) had shorter OS than other patients (Figure 2A,B,D–I, bottom curves).

However, in our study, although we did not obtain a significant difference in OS and TTR between different tumor sizes, we still could see the trend that patients with larger tumor sizes had shorter OS and TTR than those without (Figure 2C). It was also the same when we referred to the invasive depth. Tumors which infiltrated to viscera manifested the shortest TTR, then came those which infiltrated to muscles and superficial tissues (Figure 2G).

### 3.3. Univariate and Multivariate Analyses of UPS Patients

Univariate and multivariate analyses were performed to figure out independent prognostic factors of UPS patients. We observed that age (hazard ratio (HR):2.280, *p* = 0.004), tumor site (HR:1.834, *p* = 0.047), mitotic index (HR:4.553, *p* = 0.000), necrosis (HR:3.258, *p* = 0.000), Ki-67 (HR:0.553, *p* = 0.000), invasive depth (HR:2.051, *p* = 0.003), FNCLCC grade (HR:4.886, *p* = 0.001) and surgical margin (HR:2.327, *p* = 0.011) were significantly associated with OS in UPS (Table 2). Multivariate cox regression analyses identified age (HR:2.017, *p* = 0.017) and invasive depth (HR:1.772, *p* = 0.031), as independent prognostic factors for OS (Table 2).

With regard to TTR, univariate cox regression analyses identified age (HR:1931, *p* = 0.005), mitotic index (HR:4.290, *p* = 0.000), necrosis (HR:2.487, *p* = 0.000), Ki-67 (HR:0.674, *p* = 0.001), FNCLCC grade (HR:3.075, *p* = 0.000) and surgical margin (HR:2.849, *p* = 0.000) as clinicopathologic factors that correlated with TTR (Table 3). Furthermore, multivariate analyses identified that mitotic index (HR:2.993, *p* = 0.002) and surgical margin (HR:2.076, *p* = 0.010) were independent indicators for TTR (Table 3).

### 3.4. Nomogram Development and Validation

Independent prognostic parameters were incorporated to develop the nomograms for predicting 3- and 5-year OS and TTR of UPS patients (Figure 1A,B). To optimize the nomograms, we incorporated surgical margin and age in OS and TTR nomograms, respectively, so that their *p*-values in our multivariate analyses were close to 0.05 and they had been confirmed to be independent prognostic factors in the literature [21].

**Figure 3:**
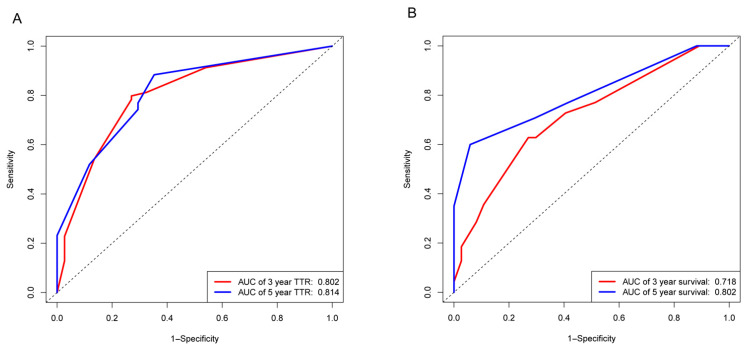
Receiver operating characteristic (ROC) curves of nomograms. (**A**) ROC curves of 3- (red line) and 5-year (blue line) TTR based on our nomogram. (**B**) ROC curves of 3- (red line) and 5-year (blue line) OS based on our nomogram. The *X*-axis represents 1-specificity. The *Y*-axis represents sensitivity.

**Figure 4:**
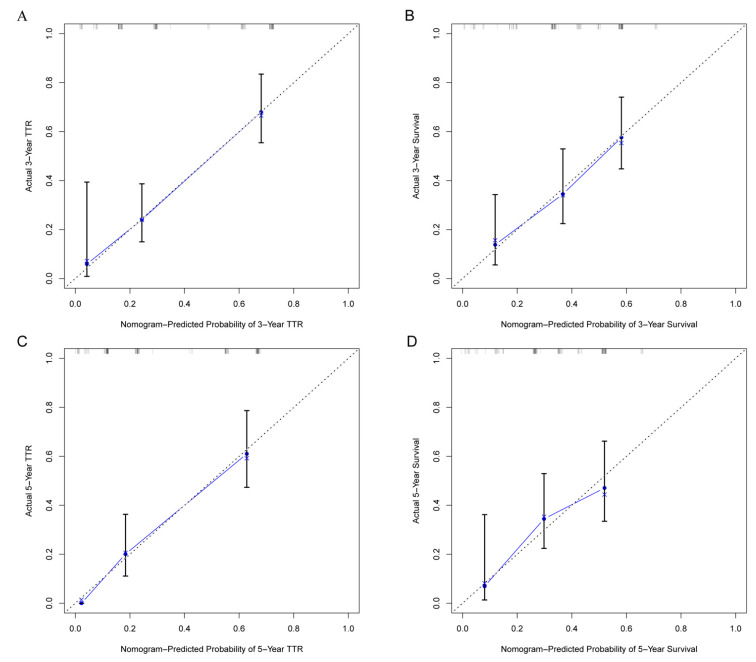
Calibration curves of predicted outcomes and actual outcomes. (**A**) 3-year and (**C**) 5-year TTR nomogram calibration curves; (**B**) 3-year and (**D**) 5-year OS nomogram calibration curves. The 45-degree line represents a perfect match between the nomogram-predicted survival (*X*-axis) and actual survival (*Y*-axis). The perpendicular line represents 95% confidence intervals of actual survival. The little bars above 1.0 actual values represent numbers of patients in a certain range.

As was shown in the nomogram, the mitotic index made the largest contribution to the TTR, followed by surgical margin and age (Figure 1A). ROC analysis showed that the Area Under Curve (AUC) of this model at 3- and 5-year TTR reached 0.802, 0.814, respectively (Figure 3A). The calibration curves demonstrated considerable consistency of predicted and actual TTR in this nomogram (Figure 4A,C).

As for OS, surgical margin made the largest contribution, then came invasive depth and age (Figure 1B). AUC of this nomogram at 3- and 5-year OS reached 0.718, 0.802, respectively (Figure 3B). Moreover, the calibration curves demonstrated considerable agreement between predicted and actual OS in this model (Figure 4B,D).

### 3.5. Risk Stratification System

**Figure 5:**
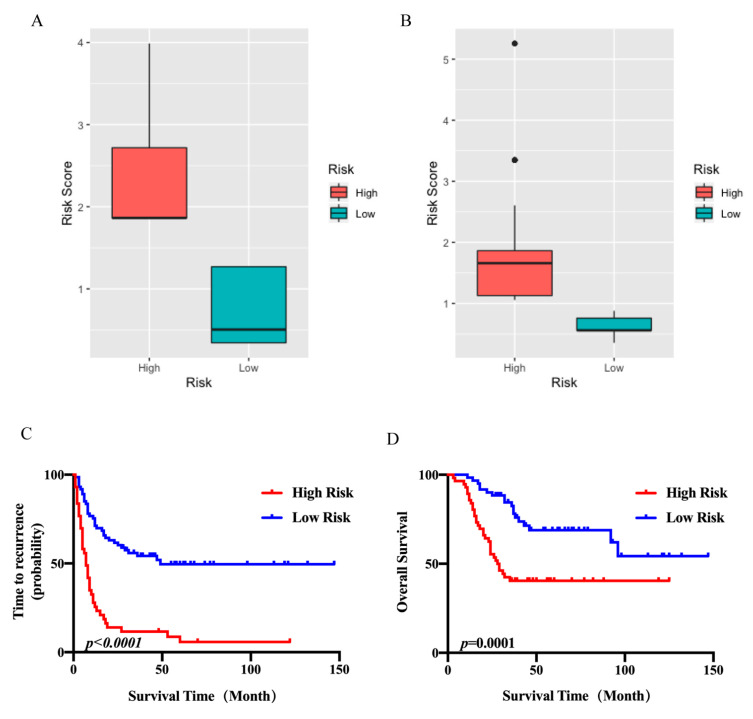
Prognostic role of risk score in UPS. (**A**) The relationship between TTR risk classifications and risk score. (**B**) The relationship between OS risk classifications and risk score. (**C**) Kaplan–Meier analysis of TTR according to TTR risk classifications. (**D**) Kaplan–Meier analysis of OS according to OS risk classifications.

Furthermore, a risk stratification system was established based on our multivariate cox regression analysis. The patients were divided into high risk and low risk groups according to the risk scores we calculated. Risk scores varied greatly between two groups (Figure 5A,B). Kaplan–Meier curves show that each risk group clearly represents a different OS and TTR (Figure 5C,D).

## 4. Discussion

UPS were highly malignant subtypes of STS accompanied by a high risk of local recurrence and distant metastasis but lacked definite directions of differentiation. All cases shared conspicuous cellular pleomorphism mixed with spindle cells and bizarre multinucleated giant tumor cells [1]. The diagnosis was made when we had excluded all the known STS, thus UPS were more likely to represent multiple sarcoma subtypes, rather than a distinct tumor entity [22]. In order to better understand the biological behaviors of UPS, we retrospectively analyzed the clinicopathological features of 116 UPS patients in our center and constructed recurrence and survival nomograms for patients who underwent surgeries for the first time.

UPS could arise throughout the body but were most commonly seen in the extremities [23]. It was consistent with our study as we had 77% of patients for whom it occurred in the extremities and trunks. A total of 73% of patients possessed tumors larger than 5 cm. It might be correlated with the malignancy of UPS as UPS cells proliferated fast or the patients’ consciousness towards health or even some socioeconomic factors such as patients in poverty being less likely to seek medical advice had an effect. The incidence of recurrence reached 65% in our study, which might represent the worse progression-free survival in the palliative stage among all histological sarcoma subtypes [19].

Local recurrence was a common phenomenon in STS. Patients with tumor recurrence were more likely to have a poor prognosis. In our study, we found that mitotic rates and surgical margin were independent factors for TTR. Inadequate margins had a statistically significant impact on local control [21]. In 1996, Le Doussal et al. analyzed 216 patients with extremity, trunk, and head/neck UPS and concluded that R2-resections were of prognostic significance while microscopic margins did not alter the outcome [4]. However, in 2015, after comprehensive analyses of 148 patients with sporadic and radiation-associated UPS of the extremities, the trunk, and the head and neck area, Dineen et al. from the MD Anderson Cancer Center found an association between R0 margins and local control (*p* = 0.019) but not OS (*p* = 0.501) [23]. Recently published literature, which presented the largest retrospective analysis for UPS with 266 patients further confirmed the accuracy of Dineen’s findings [21]. To define the optimal surgical margin, Ole et al. further analyzed the quality of microscopic negative margins and found that patients categorized with negative margin widths >5 mm had a 5-year OS rate of 92.3%, whereas patients with negative margin widths ≤1 mm displayed a 5-year OS-rate of 76.8% [24]. Although they did not obtain a statistically significant outcome, this finding might give us some inspiration to adopt a more aggressive surgical approach to attain wider negative margins. As the former literature told, our results showed that surgical margin was an independent factor for TTR but not OS, too. It was of vital importance for us in the future to figure out whether patients could benefit a lot from the expanded microscopic width of negative margins in our center. Kaplan–Meier curves showed that age, necrosis, FNCLCC classifications, and Ki-67 were also important clinicopathological factors which could influence the TTR of UPS patients.

In terms of OS, we found that age and invasive depth were independent factors for OS. Older patients had worse TTR and OS than younger patients. It might have something to do with the immune system as the immune system of older patients was worse than young patients. However, this was just our presumption. It needed further investigations to illustrate this phenomenon. Some previous studies reported that deeper tumors did not correlate to worse OS [25,26,27], while some studies showed that deeper tumors correlated with worse OS or disease-specific survival (DSS) [28,29]. However, in our study, we could see that tumors infiltrated to the viscera possessed the worst OS, followed by muscles and superficial tissues. A primary theory behind this might be that deeper infiltrated tumors had cycled through more rounds of cell division, allowing for greater outgrowth of variants which were able to infiltrate even deeper, producing metastasis. Furthermore, our results confirmed that locations, necrosis, FNCLCC classifications, Ki-67, mitosis, and surgical margin were of great importance to OS of UPS patients. Kyoungmin et al. also demonstrated that the survival outcomes were different between different tumor locations. The abdominopelvic UPS showed significantly shorter OS than UPS of other locations [30], which was in line with our results.

In the modern medical decision-making process, nomograms, which combine multiple parameters to calculate the probability of a certain event, manifested a wide application prospect [31,32,33,34]. Accumulating evidence had displayed that the nomogram showed higher prediction accuracy than other staging systems such as the American joint Committee on cancer (AJCC) staging system in multiple tumors. Therefore, it was considered as an alternative staging system [35,36,37]. Here, we constructed two novel nomograms with only three simple parameters but high accuracy for clinical doctors to predict 3- and 5-year outcomes accurately. They would be of excellent guidance to the medical decision-making process and UPS patients, who would be aware of the course of their disease in advance so that they could make preparations ahead of time.

Despite the promising result, we still had some limitations in our study. Firstly, as a result of the low morbidity of UPS, it was difficult for us to broaden our sample volume to form a validation cohort with which we could verify the accuracy and practicability of our result. Thus, prospective validation was warranted, or at least, external validation with an independent study cohort. Secondly, as a retrospective study, our level of evidence was inferior to prospective studies and we had a few biases such as selection and data missing bias, which might have an impact on our conclusions. Thirdly, our study was single-institutional so we are willing to cooperate with other medical centers to further validate the accuracy and practicability of our results.

## 5. Conclusions

In our study, we demonstrated that mitotic rates and surgical margin were independent factors for TTR while age and invasive depth were independent factors for OS. Under such circumstances, we developed two nomograms that were able to predict 3- and 5-year OS and TTR for UPS patients. These nomograms showed relatively great accuracy and practicability which might contribute a lot to the modern medical decision-making process.

## Figures and Tables

**Table 1:** Clinical characteristics of undifferentiated pleomorphic sarcoma (UPS) patients (*n* = 116).

Variables	No. (%)
Gender	
Male	56 (48%)
Female	60 (52%)
Age (years)	
<60	63 (54%)
≥60	53 (46%)
Site	
Extremities and Trunks	89 (77%)
Abdomen and others	27 (23%)
Tumor size (cm)	
<5	31 (27%)
≥5	85 (73%)
Mitosis	
Low	49 (42%)
High	67 (58%)
Necrosis	
Positive	53 (46%)
Negative	63 (54%)
Ki-67	
<50%	54 (47%)
≥50%	62 (53%)
Invasive depth	
Superficial	14 (12%)
Muscle	72 (62%)
Visceral	30 (26%)
FNCLCC grade	
G3	84 (72%)
G1 and G2	32 (28%)
Surgical margin	
Positive	21 (18%)
Negative	95 (82%)
Recurrence	
Yes	75 (65%)
No	41 (35%)
Death	
Yes	51 (44%)
No	65 (56%)

Abbreviation: FNCLCC: French Federation Nationale des Centres de Lutte Contre le Cancer.

**Table 2:** Univariate and multivariate analyses of clinicopathological features and overall survival in UPS patients.

Variable	Univariate Analysis			Multivariate Analysis		
	HR	95%CI	*p*	HR	95%CI	*p*
Gender						
Male vs. Female	0.920	0.531–1.593	0.765			
Age (years)						
<60 vs. ≥60	2.280	1.297–4.009	0.004	2.017	1.131–3.597	0.017
Site						
Extremities and Trunks vs. Abdomen and others	1.834	1.009–3.334	0.047	1.287	0.637–2.601	0.482
Tumor size (cm)						
<5 vs. ≥5	1.643	0.840–3.213	0.147			
Mitosis						
Low vs. High	4.553	2.271–9.128	0.000	1.586	0.660–3.811	0.302
Necrosis						
Positive vs. Negative	3.258	1.755–6.047	0.000	1.547	0.764–3.132	0.226
Ki-67						
<50% vs. ≥50%	0.553	0.405–0.754	0.000	0.800	0.561–1.142	0.219
Invasive depth						
Superficial vs. Muscle vs. Visceral	2.051	1.274–3.301	0.003	1.772	1.054–2.981	0.031
FNCLCC grade						
G3 vs. G1 and G2	4.886	1.934–12.343	0.001	2.050	0.711–5.914	0.184
Surgical margin						
Positive vs. Negative	2.327	1.209–4.478	0.011	1.850	0.918–3.727	0.085
SMA						
Positive vs. Negative	1.045	0.782–1.395	0.767			

Abbreviation: FNCLCC: French Federation Nationale des Centres de Lutte Contre le Cancer, SMA: Smooth Muscle Actin, HR: Hazard ratio, CI: Confidence interval. Cox regression analyses were performed for all the above data. Missing values meant that variables were not put into multivariate analysis as they did not reach statistically significance in univariate analysis.

**Table 3:** Univariate and multivariate analyses of clinicopathological features and time to recurrence in UPS patients.

Variable	Univariate Analysis			Multivariate Analysis		
	HR	95%CI	*p*	HR	95%CI	*p*
Gender						
Male vs. Female	1.124	0.714–1.772	0.631			
Age (years)						
<60 vs. ≥60	1.931	1.221–3.053	0.005	1.456	0.910–2.331	0.117
Site						
Extremities and Trunks vs. Abdomen and others	1.075	0.632–1.827	0.791			
Tumor size (cm)						
<5 vs. ≥5	1.561	0.895–2.723	0.116			
Mitosis						
Low vs. High	4.290	2.503–7.355	0.000	2.993	1.482–6.046	0.002
Necrosis						
Positive vs. Negative	2.487	1.529–4.046	0.000	1.047	0.585–1.875	0.877
Ki-67						
<50% vs. ≥50%	0.674	0.532–0.854	0.001	0.920	0.701–1.207	0.547
Invasive depth						
Superficial vs. Muscle vs. Visceral	1.389	0.964–2.002	0.078			
FNCLCC grade						
G3 vs. G1 and G2	3.075	1.647–5.741	0.000	1.207	0.557–2.612	0.634
Surgical margin						
Positive vs. Negative	2.849	1.682–4.828	0.000	2.076	1.193–3.611	0.010
SMA						
Positive vs. Negative	1.327	0.807–2.183	0.265			

Abbreviation: FNCLCC: French Federation Nationale des Centres de Lutte Contre le Cancer, SMA: Smooth Muscle Actin, HR: Hazard ratio, CI: Confidence interval. Cox regression analyses were performed for all the above data. Missing values meant that variables were not put into multivariate analysis as they did not reach statistically significance in univariate analysis.

## Data Availability

The data presented in this study are available on request from the corresponding author. The data are not publicly available due to privacy.
